# The Elephant and the Spandrel

**DOI:** 10.1093/emph/eoae019

**Published:** 2024-08-26

**Authors:** Zachary T Compton, J Arvid Ågren, Andriy Marusyk, Aurora M Nedelcu

**Affiliations:** University of Arizona Cancer Center, Tucson, AZ, USA; University of Arizona College of Medicine, Tucson, AZ, USA; Lerner Research Institute, Cleveland Clinic Foundation, Cleveland, OH, USA; Department of Evolutionary Biology, Uppsala University, Uppsala, Sweden; Department of Metabolism and Physiology, H. Lee Moffitt Cancer Center and Research Institute, Tampa, FL, USA; Department of Biology, University of New Brunswick, Fredericton, Canada

## Abstract

Comparative oncology has made great strides in identifying patterns of cancer prevalence and risk across the tree of life. Such studies have often centered on elucidating the evolution of mechanisms that prevent the development and progression of cancer, especially in large animals such as elephants. Conclusions from this approach, however, may have been exaggerated, given that the deep evolutionary origins of multicellularity suggest that the preeminent functions of the identified mechanisms may be unrelated to cancer. Instead, cancer suppression may have emerged as an evolutionary byproduct, or “spandrel”. We propose a novel evolutionary perspective that highlights the importance of somatic maintenance as the underlying axis of natural selection. We argue that by shifting the focus of study from cancer suppression to somatic maintenance, we can gain a deeper understanding of the evolutionary pressures that shaped the mechanisms responsible for the observed variation in cancer prevalence across species.

## INTRODUCTION

Comparative oncology studies, i.e. comparing cancer incidence across the tree of life and identifying specific genomic determinants of differences in cancer risk, provide unique insights on cancer biology and assist with the discovery of new cancer prevention mechanisms [1–3]. The success of these studies, however, is at least partially dependent on the accuracy of the interpretation of the observed differences. The discovery of species with substantially lower cancer rates led to the conclusion that genetic mechanisms that directly influence cancer suppression have been selected specifically for this function; that is, in these species, cancer risk poses a strong selective pressure that resulted in the evolution of additional/better specific anti-cancer mechanisms. The paradigm that these cancer-suppression mechanisms evolved specifically for this role defines the key assumptions in interpreting the observed genetic differences between species with different cancer rates, as these differences are assumed to directly be responsible for cancer incidence.

**Figure 1.**
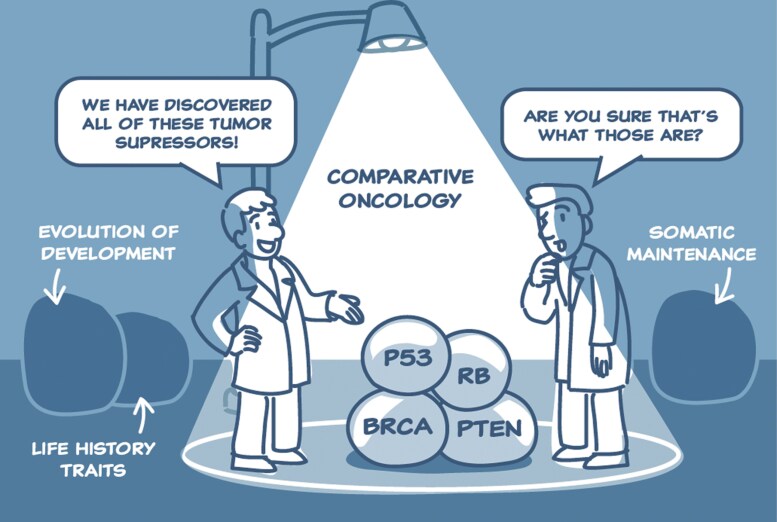
The current paradigm in comparative oncology (designed by Sabine Deviche)

We argue that decreased cancer rates need not necessarily be a result of a direct selection for mechanisms specifically/uniquely involved in tumor suppression. Instead, we suggest a more parsimonious connection, where selection acts on a more general trait of enhanced tissue maintenance in species with slower life histories (i.e. larger body mass and longer lifespan) (Fig. 1). Under this argument, reduced cancer rates are a consequence of this direct selection on a trait that has indirect and strong multiple effects on fitness, including (but not limited) to cancer suppression. This distinction has significant practical implications. If our argument on the indirect selection on cancer suppression is correct, therapeutic interventions that rejuvenate tissues and prevent aging-associated functional decline may have beneficial side effects of also decreasing the odds of incidence of the deadly disease [4–6].

## THE ADAPTATIONIST PARADIGM

Adaptation, George C. Williams noted in *Adaptation and Natural Selection*, is ‘a special and onerous concept that should not be used unnecessarily’ [7]. An organismal trait should be called an adaptation if and only if we can demonstrate that it is a product of natural selection, rather than a chance event. At the time, Williams was particularly concerned with the prevalence of naïve group selection arguments, the lazy inference of selection at the level of the group without proper evidence. A decade and a half later, Gould and Lewontin had a bigger goal when they attacked what they called ‘adaptive stories’ in their ‘The spandrels of San Marco and the Panglossian paradigm: a critique of the adaptationist programme’ [8]. Much-cited and much debated, the paper gave birth to the spandrels metaphor, to contrast with an *a priori* reliance on selective explanations of traits and to highlight the failure to consider alternatives, such as historical contingencies, developmental constraints, or selection on correlated traits. While the power of Williams’s argument has been recognized by both friends [9] and foes [10] of group selection, the merits of the Gould and Lewontin intervention have been contested. Indeed, the dispute has involved some of the testier exchanges in the recent history of evolutionary biology (see, e.g. the exchange between Dennett and Lewontin in *Behavioral and Brain Sciences* [11, 12] and between Dennett and Gould in *The New York Review of Books* [13, 14]).

Since then, terms like ‘adaptationist research program’ and ‘adaptationism’ have been shown to have many meanings [15, 16]. As a field, Evolutionary Medicine has often relied on what can be called a methodological kind, where adaptation acts as the organizing principle around which to ask biological questions. Take the founders of the field, Williams and Nesse, who in their classic 1991 review stated: ‘When confronted with a biological phenomenon, try to envisage it as an aspect of an adaptation’ [17] p.3. In the 30 years since the approach has paid dividends. For example, assuming that a fever is adaptive in the context of an infection is useful information for patients and medical providers in determining when a patient should let a fever take its course [18]. Similarly, Nesse’s theoretical contribution on the evolutionary origin of psychiatric maladies has been demonstrated to be a powerful tool in a field with otherwise waning theoretical foundations [19–21].

The considerable success the adaptationist program has had within Evolutionary Medicine does not mean it is infallible when applied to all diseases. A key lesson of the spandrels metaphor was the importance of distinguishing historical origin from current utility [14]. Our interpretations of evolutionary processes that shaped vertebrate biology dictate our capacity to identify similar processes elsewhere on the tree of life. Yet, the ability to make such distinctions remains a problem in evolutionary biology, and it appears to be an especially penetrative one within Evolutionary Medicine. This becomes especially important as the field moves from describing human behavior and physiology to investigating the role of genomic mechanisms in disease risk. In this paper, we argue that comparative oncology has put too much emphasis on putative novel cancer-suppression mechanisms (assumed/implied to have evolved specifically for this function) that can actually be better explained by their general evolutionary role in improved somatic maintenance (defined here as including all the processes and mechanisms that ensure the functionality of soma, including DNA damage-induced apoptosis, autophagy, immune surveillance, purifying selection—see [22]).

## THE ADAPTATIONIST PROGRAM IN COMPARATIVE ONCOLOGY

The adaptationist view of disease conceptualizes specific aspects as adaptive defenses (e.g. fever) or reflections of historically adaptive responses (e.g. panic attacks) and has permeated several subfields. One example is comparative oncology, the emergence of which combined with the increasing availability of cross-species pathology data has verified the ubiquity of cancer-like processes across the tree of life [23–26]. The striking variation in observed cancer prevalence across vertebrates means that comparative oncology is a field ripe for explorations and many hypotheses have been put forward for what explains species-level vulnerabilities or resistance [24–26]. In particular, much emphasis has been put on mechanisms that suppress the proliferation of tumors [1, 27–33]. However, we argue that we should avoid conflating differences in the ubiquity of the phenotype (cancer prevalence) with evidence for direct selection on mechanisms to constrain the phenotype (cancer suppression). To interpret the existence of mechanisms that may be associated with enhanced cancer suppression as an assumption that they evolved explicitly for this purpose is an unfortunate example of affirming the consequent.

In contrast with most other phenotypes studied by the comparative method, cancer is not a heritable trait (although genes associated with susceptibility to cancer can be transmitted and maintained in populations) [34]. Nevertheless, the observed differences in cancer incidence among individuals, populations and species will have been shaped by evolution. Scholars in the field have generally fallen into the camps of interpreting these differences in terms of either resistance or vulnerability to cancer. The resistance camp invokes differences in adaptive mechanisms that evolved in response to selection against cancer or as byproducts of selection on other traits [3, 30, 35–38]. On the other hand, the vulnerability camp envisions that cancer is the consequence of either evolutionary trade-offs or the decline in the strength of selection with age, and cancer incidence reflects differences in life-history trade-offs like reproduction *versus* somatic maintenance, genes with antagonistic pleiotropic effects, evolutionary mismatch and recent life history changes (such as the recent increase in lifespan in the human lineage and the domestication of animals) [39–41]. Here, we are arguing that differences in cancer incidence reflect differences in selection on traits that affect development and somatic maintenance, with cancer resistance being a byproduct. The specific claim for the importance of cancer suppression has been bolstered by comparative work. For example, Brown *et al*. [23] and Aktipis *et al*. [42] both argue that the deep phylogenetic roots of cancer-suppression genes suggest the importance of cancer risk as a selective force. Another key study in the history of this argument is Domazet-Loso and Tautz’s [43] highly influential paper that used a method that relates protein families to major evolutionary events (phylostratigraphy) to date the origins of cancer-related genes to the dawn of the metazoans. However, as Domazet-Loso and Tautz themselves note, there are nuances in how to interpret their data. Most importantly, the gene set they classified as the cancer-related protein domains had associations not only to the origin of metazoans but also to the origin of self-contained cells. Yet, it is the former association that has been emphasized and taken as evidence for the importance of specific cancer-suppression genes and mechanisms.

## HOW WE ENDED UP HERE

A central reason for the wide acceptance of the evolved cancer-suppression paradigm lies in the history of biomedical research. Cancer research has the lion’s share of all the funding that historically supported and currently supports biomedical research [44]. Many of the genes that influence cancer incidence have been discovered by correlation analyses and experimental studies explicitly aimed at finding the genetic determinants of cancer, which is reflected in their names. Depending on the species in which the disruption was first observed, this naming convention can have implications for the hypotheses investigated when studying other lineages. Take, for example, the BRCA1/2 genes, the intensely studied pair known as Breast Cancer Susceptibility Genes largely famous for their mutant germline variant responsible for familial cases of breast cancer [45–47]. The identification of their key role in lifetime breast cancer risk has saved innumerable lives. This important contribution notwithstanding, BRCA1/2’s definition as a ‘breast cancer gene’ has relegated it to be studied nearly exclusively in the context of cancer.

Individual genes, however, rarely, if ever have a single predefined function. Instead, individual genes are part of gene regulatory networks that typically control multiple biological processes. Furthermore, genes likely have different functions in different species, such that inferring ancestral functions based on one involvement in humans may be misleading. BRCA1/2 homologs have been found across nearly all domains of life, with an origin pointing to the emergence of self-containing cells [48]. They appear to have roles in activities unrelated to cancer (Table 1). Have we overlooked interesting questions on the evolution and function of this gene by classifying it as a breast cancer gene? What, for example, is the use of a breast cancer gene in fungi [67]? This problem of gene naming has been exacerbated for those pursuing evolutionary studies of gene origins and phylostratigraphy. For researchers studying ‘cancer’ genes in single-celled species, to what extent have we diminished their ability to form hypotheses by prioritizing their relevance to cancer? And to what extent are we making inferences about the evolutionary origin of the function (i.e. cancer suppression) associated with the human protein?

**Table 1.** The main ‘tumor suppressor genes’, their phylogenetic distribution, and the main corresponding protein functions with their roles in cancer-related and -unrelated activities

Gene name	Phylogenetic distribution (homologs)	Protein function	Cancer-suppression activities	Cancer-unrelated activities
TP53	Metazoa Choanoflagellates	Transcription factor	Cell cycle regulation, DNA damage repair, programmed cell death [49]	Development (mice and zebrafish) [50, 51], wound healing and regeneration (planarians) [52], tissue homeostasis and metabolic adaptation (*Drosophila*) [53], stress-induced apoptosis in germline (flies and nematodes) [54]
BRCA1	Metazoa Land plants Green algae	Recruits repair proteins to DNA damage sites	Cell cycle regulation and maintenance of chromosomal integrity [55, 56]	Development (animals) [54]
BRCA2	Metazoa Land plants Green algae Fungi	Recruits repair proteins to DNA damage sites	Cell cycle regulation and maintenance of chromosomal integrity [55, 56]	Meiosis (vertebrates, fungi and plants) [57–59], systemic acquired resistance in plants [60]
PTEN	Metazoa Land plants Green algae Fungi	Dual phosphatase with both protein and lipid phosphatase activities	Cell migration, cell growth, DNA damage repair, cell survival signaling [61]	Metabolic regulator (e.g. glycolysis, gluconeogenesis, glycogen synthesis, lipid metabolism, mitochondrial metabolism) in animals [62]. Effector of lipid signaling (plants) [63]. Defense mechanisms, sporulation and virulence (fungi) [64].
Rb	Metazoa Land plants Green algae	Transcription factor	Cell cycle regulation [65]	Development (e.g. asymmetric cell division, stem cell maintenance and DNA damage response) in plants [66].

## TAKING DEVELOPMENT SERIOUSLY

The currently accepted view that cancer is a disease of multicellularity often comes with the associated assumption that the ability to control cell proliferation and differentiation is a trait that evolved specifically in multicellular lineages. However, the capacity to control cell proliferation and differentiation in response to environmental factors is important for all unicellular organisms (see Nedelcu [68] for discussion). Furthermore, as noted above, differences in cancer prevalence among lineages are usually interpreted in terms of differences in cancer-suppression mechanisms [2, 29, 30, 69, 70]. Yet, many of these—such as tissue organization and architecture, stem cell dynamics, cell competition—are also associated with normal developmental mechanisms and processes. However, the development and architecture of animal tissues are often assumed to reflect an organism’s need to limit cancer [3]. By this argument, cancer shaped the evolution of development [71]. An alternative interpretation is that it is the other way around: the vulnerability or resistance to cancer is the outcome of developmental processes that evolved in response to (or as a byproduct of) specific life-history traits or selective pressures unrelated to cancer. In this scenario, the evolution of development can shape cancer and the difference in the so-called ‘cancer suppression mechanisms’ in fact reflects differences in processes and pressures unrelated to cancer [71]. Examples would include the selection of other critical and life-sustaining functions such as germline quality control, development and somatic maintenance.

Indeed, whereas the evolution of mechanisms that prevent mutations and uncontrolled cell proliferation is generally discussed in the context of cancer suppression, reducing the impact of mutations and regulation of cell proliferation are also vital during embryonic development, a stage during which selection typically acts strongly [72]. In line with this, most of the cancer-suppression mechanisms are also involved in normal development and their deregulation will affect fitness in many cancer-unrelated ways [73] (Table 1). Consequently, their maintenance is likely strongly influenced by these cancer-unrelated roles; such as mutations in these genes will also affect fitness early in life (i.e. these genes have pleiotropic effects).To consider cancer as the main selective pressure that shaped their evolution therefore implies that cancer threat is more significant than all other fitness-affecting developmental processes combined.

## THE PRIMACY OF SOMATIC MAINTENANCE

Aging is the main factor associated with increased vulnerability to cancer [3, 74]. Cancers in old age are often interpreted as the expected consequence of the decline of the strength of selection on cancer suppression mechanisms during post-reproductive life [74]. However, because of their general roles in development, the so-called cancer-suppression mechanisms are in fact contributing to somatic maintenance themselves. That is, mechanistically, these cancer suppression mechanisms are nothing but somatic maintenance mechanisms. In this view, cancer is not an outcome of aging. Rather, age-related cancer is a component (not an additional consequence) of the aging phenotype associated with the deterioration of soma. The latter can be due to the decline of the strength of selection on somatic maintenance with age and/or reproduction-survival trade-offs [39]. Differences in cancer incidence among lineages therefore reflect differences in selection for somatic maintenance, not for cancer suppression. Variation in the strength of selection for somatic maintenance, in turn, is due to differences in extrinsic mortality and/or survival-reproduction trade-offs that can affect lifespan and reproductive decline with advancing age. Again, not because of differences in cancer risk, such as those associated with an increased number of cells and cell divisions in large, long-lived animals, like elephants.

Evidence in favor of this interpretation is the fact that several cancer hallmarks are also aging hallmarks [75] (Fig. 6 in Nedelcu [71]), suggesting that cancer and aging share common proximate causes. Accounting for this possibility forces us to switch the focus away from the evolution of specific cancer-suppression mechanisms and toward understanding the selective factors that drive increased investments in somatic maintenance (which will directly and indirectly suppress cancer), and the evolved mechanisms associated with the recently coined somatic maintenance program [22]. In addition to the evidence highlighted here, mathematical modeling supports the argument that the strength of the somatic maintenance program can modulate cancer risk; a strong soma will slow or prevent the growth of oncogenic clones, while a weak soma will favor oncogenesis [22]. In other words, differences in cancer risk are byproducts of differences in somatic maintenance.

## THE EVOLUTIONARY ROLES OF SOMATIC MAINTENANCE

Evolutionary game theory has provided a salient framework to understand the emergence of multicellularity as driven by the evolution drivers of mechanisms that ensure and enforce cell-cell cooperation at the emergence of multicellularity [23, 76, 77]. Comparative oncology has drawn heavily from these studies to suggest the evolutionary origins of tumor suppression [23, 76, 77]. These conceptual arguments typically rely on referring to primitive systems of cancer suppression as systems of ‘cheater detection’, where cellular cheating is defined as any single cell that betrays the foundations of multicellularity [23]. Despite the utility of this analogy, it is equally valid to describe the necessity of cheater suppression at the dawn(s) of multicellularity in terms of maintaining all somatic functions. In this framework, the transitions from single-celled life to multicellularity were facilitated by the emergence of regulatory mechanisms that would retain the cellular phenotypes that distinguished them from their single-celled ancestors [23, 76, 78–83]. In particular, mechanisms of separating the germ from somatic cells and then preserving the integrity, stability and functionality of somatic cells all fall under the broad characterization of ‘somatic maintenance’. The robustness of these mechanisms of multicellular control, alongside the emergence of more sophisticated ones in response to specific selective pressures (e.g. increased body size/number of somatic cells and/or lifespan), would have been an overwhelming selective advantage within the evolving populations of multicellular species. While the disruption in many of these cellular signaling networks could have likely led to cellular phenotypes that we commonly ascribe to cancer, they would have had negative fitness effects far more proximal to an organism’s survival than the formation of tumors, especially affecting embryonic development and early maturation in these species. For instance, an increased number of mutations—which would be expected in large animals, would first negatively affect the processes associated with embryogenesis and development of a large body size (which require a larger number of cell divisions). Thus, additional mechanisms to prevent the accumulation of mutations would be required to ensure proper development and early maturation of large body sizes. Consequently, selection on these processes would favor mechanisms that could also contribute to cancer suppression later in life.

Several papers, notably Erten and Kokko [84] and Boddy *et al*. [85], have developed mathematical models to trace out the potential evolutionary trajectories for multicellular species to mitigate the risk of cancer development across different life histories. The core of the discussion in these papers hits at the crux of our argument. Which is that these evolutionary trajectories would have had to have played out in mitigating the risks of deteriorating cellular cooperation with or without the specific risk of cancer mortality.

## SEARCHING UNDER A LAMPPOST

There has been no shortage of discoveries in comparative oncology that propose new mechanisms for cancer suppression in species with exceptionally low cancer prevalence, such as the elephant [1, 29, 32, 86–88]. Clarifying the origin, evolution and current function of these ‘cancer suppression genes’ requires an understanding of both proximate and ultimate causes [89, 90].To debate whether these uncovered mechanisms, or enhancement of existing mechanisms, are uniquely cancer suppressive (i.e. evolved specifically for this function, in response to increased cancer risk) or rather more general mechanisms of somatic maintenance is therefore not purely semantics. How we define terms and categorize functions in comparative genomics sets the parameters for what we look for.

Isolating genes, gene sets or even entire signaling networks for their unique involvement in cancer processes has already sparked some debate in the cancer biology field. The term ‘anti-cancer mechanism’ has gone the way of cancer-related genes [91]: the more we look, the more everything is or can be said to be involved. The narrative in publications that discuss potential anti-cancer mechanisms are often reliant on the apparent necessity (though often just based on correlations) of these mechanisms in exceptionally long-lived species [30, 31, 33, 86, 88, 92, 93]. However, cell cycle control, DNA damage repair, immune surveillance and other examples of putative anti-cancer pathways are all classes that we expect selection to enhance their robustness to scale with lifespan and size regardless of cancer risk. Consider the following thought experiment. Which of the above mechanisms, often attributed as anti-cancer, would a species *not* need in the absence of a cancer threat? Would genes such as p53, Rb or BRCA not have been selected if cancer had *no* major effect on fitness? For instance, plants, due to lack of cell migration and vital organs, do not experience the same negative fitness effects that cancer can have in animals. Yet, they do possess mechanisms (and genes such as Rb and BRCA) that ensure proper cell proliferation and elimination of damaged cells. Moreover, by classifying these mechanisms as cancer suppressive, do we blind ourselves to their role in other processes unrelated to cancer and their relevance to other diseases, especially those related to other somatic dysfunctions?

## CONCLUSION

The wider integration of evolutionary theory with modern medicine is primed to make discoveries that transform many of our preconceived notions on the origins of disease and their risk factors. Our ability to translate these discoveries into clinical practices, however, depends on our ability to correctly identify true adaptations. Just like how an over-reliance on adaptive scenarios can lead us astray, so can too much focus on constraint lead to similar kinds of just-so-stories ([94], p. 20). As we expand our efforts to determine the role that genomic mechanisms have in determining disease risk, we need to be careful to tell the two apart.

## References

[1] Vazquez JM , Sulak M, Chigurupati S et al A zombie LIF gene in elephants is upregulated by TP53 to induce apoptosis in response to DNA damage. Cell Rep 2018;24:1765–76. 30110634 10.1016/j.celrep.2018.07.042

[2] Abegglen LM , Caulin AF, Chan A et al Potential mechanisms for cancer resistance in elephants and comparative cellular response to DNA damage in humans. JAMA 2015;314:1850–60. 26447779 10.1001/jama.2015.13134 PMC4858328

[3] DeGregori J. Evolved tumor suppression: why are we so good at not getting cancer? Cancer Res 2011;71:3739–44. 21610109 10.1158/0008-5472.CAN-11-0342 PMC3677553

[4] Somarelli JA , Rupprecht G, Altunel E et al A comparative oncology drug discovery pipeline to identify and validate new treatments for osteosarcoma. *Cancers* 2020;12. DOI: https://doi.org/10.3390/cancers12113335. PMC7696249 33187254

[5] Somarelli JA , Boddy AM, Gardner HL et al Improving cancer drug discovery by studying cancer across the tree of life. Mol Biol Evol 2020;37:11–7. 31688937 10.1093/molbev/msz254 PMC8204703

[6] Rao SR , Somarelli JA, Altunel E et al From the clinic to the bench and back again in one dog year: how a cross-species pipeline to identify new treatments for sarcoma illuminates the path forward in precision medicine. Front Oncol 2020;10. https://www.frontiersin.org/articles/10.3389/fonc.2020.00117. 10.3389/fonc.2020.00117 PMC7026496 32117764

[7] Williams GC , Burt A. *Williams: Adaptation and Natural Selection [Internet]*. sscnet.ucla.edu; 1997. https://www.sscnet.ucla.edu/comm/steen/cogweb/Abstracts/Williams_66.html (23 Aug 2023, date last accessed).

[8] Gould SJ , Lewontin RC. The spandrels of San Marco and the Panglossian paradigm: a critique of the adaptationist programme. Proc R Soc Lond B Biol Sci 1979;205:581–98. 42062 10.1098/rspb.1979.0086

[9] Sober E , Wilson DS. Adaptation and natural selection revisited. J Evol Biol 2011;24:462–8. 21226890 10.1111/j.1420-9101.2010.02162.x

[10] Boomsma JJ. Fifty years of illumination about the natural levels of adaptation. Curr Biol 2016;26:R1250–5. 27997830 10.1016/j.cub.2016.11.034

[11] Dennett DC. Intentional systems in cognitive ethology: the “Panglossian paradigm” defended. Behav Brain Sci 1983;6:343–55.

[12] Lewontin RC. Elementary errors about evolution. Behav Brain Sci 1983;6:367–8.

[13] Dennett D. ”Darwinian Fundamentalism’: an exchange. New York Rev Books [Internet] 1997;44. https://philpapers.org/rec/DENDFA.

[14] Gould SJ. Darwinian fundamentalism. New York Rev Books 1997;44:34–7.

[15] Lewens T. Seven types of adaptationism. Biol Philos 2009;24:161–82.

[16] Godfrey-Smith P. Three kinds of adaptationism. Adapt Optimal 2001;122:335–57.

[17] Williams GC , Nesse RM. The dawn of Darwinian medicine. Q Rev Biol 1991;66:1–22. 2052670 10.1086/417048

[18] Wrotek S , LeGrand EK, Dzialuk A et al Let fever do its job: the meaning of fever in the pandemic era. Evol Med Public Health 2021;9:26–35. 33738101 10.1093/emph/eoaa044 PMC7717216

[19] Nesse RM. *Good Reasons for Bad Feelings: Insights From* *the Frontier Of Evolutionary Psychiatry* . 2019. [Accessed September 2023] https://books.google.ca/books?hl=en&lr=&id=frmHDwAAQBAJ&oi=fnd&pg=PR13&ots=TBHFiHMFwl&sig=4usb1AswcmAYa-x4Ex8z_v5i9m8

[20] Nesse RM. *Why Evolutionary Do Mental Disorders Persist? Perspectives on Evolution and Mental Health [Internet]* . 2022. [Accessed September 2023] https://books.google.com/books?hl=en&lr=&id=Tph-EAAAQBAJ&oi=fnd&pg=PA84&dq=Why+Evolutionary+Mental+Disorders+Persist&ots=d6KaREPMi8&sig=bnsD-2uUjZKVB6BjEd76YEgWgB0

[21] Nesse RM , Ellsworth PC. Evolution, emotions, and emotional disorders. Am Psychol 2009;64:129–39. 19203145 10.1037/a0013503

[22] Rozhok A , DeGregori J. Somatic maintenance impacts the evolution of mutation rate. BMC Evol Biol 2019;19:172. 31443631 10.1186/s12862-019-1496-y PMC6708161

[23] Aktipis CA , Boddy AM, Jansen G et al Cancer across the tree of life: cooperation and cheating in multicellularity. Philos Trans R Soc Lond B Biol Sci [Internet] 2015;370:20140219. DOI: https://doi.org/10.1098/rstb.2014.0219 26056363 PMC4581024

[24] Bulls SE , Platner L, Ayub W et al Cancer Prevalence Is Related to Body Mass and Lifespan in Tetrapods and Remarkably Low in Turtles [Internet]. In *bioRxiv*. 2023. p. 2022.07.12.499088. DOI: https://doi.org/10.1101/2022.07.12.499088

[25] Compton Z , Harris V, Mellon W et al *Cancer Prevalence Across Vertebrates* . Cancer Discov. 2025;15:227–244. DOI: https://doi.org/10.1158/2159-8290.CD-24-0573 PMC11726020 39445720

[26] Vincze O , Colchero F, Lemaître JF et al Cancer risk across mammals. Nature 2022;601:263–7. 34937938 10.1038/s41586-021-04224-5 PMC8755536

[27] Chiari Y , Glaberman S, Lynch VJ. Insights on cancer resistance in vertebrates: reptiles as a parallel system to mammals. Nat Rev Cancer 2018;18:525. 29891962 10.1038/s41568-018-0033-4

[28] Vazquez JM , Pena MT, Muhammad B et al Parallel evolution of reduced cancer risk and tumor suppressor duplications in Xenarthra. Elife [Internet] 2022;11:e82558. DOI: https://doi.org/10.7554/eLife.82558 36480266 PMC9810328

[29] Sulak M , Fong L, Mika K et al TP53 copy number expansion is associated with the evolution of increased body size and an enhanced DNA damage response in elephants. Elife [Internet] 2016;5:5. DOI: https://doi.org/10.7554/eLife.11994 PMC5061548 27642012

[30] Tollis M , Boddy AM, Maley CC. Peto’s Paradox: how has evolution solved the problem of cancer prevention? BMC Biol 2017;15:60. 28705195 10.1186/s12915-017-0401-7 PMC5513346

[31] Tollis M , Schneider-Utaka AK, Maley CC. The evolution of human cancer gene duplications across mammals. Mol Biol Evol 2020;37:2875–86. 32421773 10.1093/molbev/msaa125 PMC7530603

[32] Tollis M , Robbins J, Webb AE et al Return to the sea, get huge, beat cancer: an analysis of cetacean genomes including an assembly for the Humpback Whale (Megaptera novaeangliae). Mol Biol Evol 2019;36:1746–63. 31070747 10.1093/molbev/msz099 PMC6657726

[33] Tollis M , Schiffman JD, Boddy AM. Evolution of cancer suppression as revealed by mammalian comparative genomics. Curr Opin Genet Dev 2017;42:40–7. 28161621 10.1016/j.gde.2016.12.004

[34] Fernandez AA. A cancer-causing gene is positively correlated with male aggression in Xiphophorus cortezi. J Evol Biol 2010;23:386–96. 20021547 10.1111/j.1420-9101.2009.01914.x PMC2901164

[35] Nunney L. Resolving Peto’s paradox: modeling the potential effects of size-related metabolic changes, and of the evolution of immune policing and cancer suppression. Evol Appl 2020;13:1581–92. 32821274 10.1111/eva.12993 PMC7428811

[36] Brown JS , Cunningham JJ, Gatenby RA. The multiple facets of Peto’s paradox: a life-history model for the evolution of cancer suppression. Philos Trans R Soc Lond B Biol Sci [Internet] 2015;370:20140221. DOI: https://doi.org/10.1098/rstb.2014.0221 26056365 PMC4581026

[37] Kapsetaki SE , Compton ZT, Mellon W et al Germline mutation rate predicts cancer mortality across 37 vertebrate species. *Evolution, Medicine, and Public Health* 2024;12:122–8. 39233763 10.1093/emph/eoae016 PMC11372239

[38] Kapsetaki SE , Basile AJ, Compton ZT et al The relationship between diet, plasma glucose, and cancer prevalence across vertebrates. Nat Communications 2025;16:1–11. 10.1038/s41467-025-57344-1 PMC11904020 40074744

[39] Lemaître JF , Pavard S, Giraudeau M et al Eco‐evolutionary perspectives of the dynamic relationships linking senescence and cancer. Funct Ecol 2020;34:141–52.

[40] Dujon AM , Boutry J, Tissot S et al Cancer susceptibility as a cost of reproduction and contributor to life history evolution. Front Ecol Evol [Internet] 2022;10. https://www.frontiersin.org/articles/10.3389/fevo.2022.861103.

[41] Aktipis CA , Nesse RM. Evolutionary foundations for cancer biology. Evol Appl 2013;6:144–59. 23396885 10.1111/eva.12034 PMC3567479

[42] Brown JS , Aktipis CA. Inclusive fitness effects can select for cancer suppression into old age. Philos Trans R Soc Lond B Biol Sci [Internet] 2015;370. DOI: https://doi.org/10.1098/rstb.2015.0160 PMC4581037 26056358

[43] Domazet-Loso T , Tautz D. Phylostratigraphic tracking of cancer genes suggests a link to the emergence of multicellularity in metazoa. *BMC Biology* 2010;8:66. 20492640 10.1186/1741-7007-8-66 PMC2880965

[44] National Institutes of Health (NIH) [Internet]. 2015. Appropriations (Section 1). https://www.nih.gov/about-nih/what-we-do/nih-almanac/appropriations-section-1 (21 July 2024, date last accessed).

[45] Tutt A , Ashworth A. The relationship between the roles of BRCA genes in DNA repair and cancer predisposition. Trends Mol Med 2002;8:571–6. 12470990 10.1016/s1471-4914(02)02434-6

[46] Venkitaraman AR. Linking the cellular functions of BRCA genes to cancer pathogenesis and treatment. Annu Rev Pathol 2009;4:461–87. 18954285 10.1146/annurev.pathol.3.121806.151422

[47] Gorodetska I , Kozeretska I, Dubrovska A. BRCA genes: the role in genome stability, cancer stemness and therapy resistance. J Cancer 2019;10:2109–27. 31205572 10.7150/jca.30410 PMC6548160

[48] Pfeffer CM , Ho BN, Singh ATK. The evolution, functions and applications of the breast cancer genes BRCA1 and BRCA2. Cancer Genom Proteom 2017;14:293–8. 10.21873/cgp.20040 PMC5611516 28870997

[49] Sabapathy K , Lane DP. Understanding p53 functions through p53 antibodies. J Mol Cell Biol 2019;11:317–29. 30907951 10.1093/jmcb/mjz010 PMC6487784

[50] Vousden KH , Lane DP. p53 in health and disease. Nat Rev Mol Cell Biol 2007;8:275–83. 17380161 10.1038/nrm2147

[51] Choi J , Donehower LA. p53 in embryonic development: maintaining a fine balance. Cell Mol Life Sci 1999;55:38–47. 10065150 10.1007/s000180050268 PMC11146796

[52] Sánchez Alvarado A. Cellular hyperproliferation and cancer as evolutionary variables. Curr Biol 2012;22:R772–8. 22975008 10.1016/j.cub.2012.08.008 PMC3590310

[53] Ingaramo MC , Sánchez JA, Dekanty A. Regulation and function of p53: a perspective from Drosophila studies. Mech Dev 2018;154:82–90. 29800619 10.1016/j.mod.2018.05.007

[54] Sutcliffe JE , Brehm A. Of flies and men; p53, a tumour suppressor. FEBS Lett 2004;567:86–91. 15165898 10.1016/j.febslet.2004.03.122

[55] Welcsh PL , Owens KN, King MC. Insights into the functions of BRCA1 and BRCA2. Trends Genet 2000;16:69–74. 10652533 10.1016/s0168-9525(99)01930-7

[56] Foulkes WD , Shuen AY. In brief: BRCA1 and BRCA2. J Pathol 2013;230:347–9. 23620175 10.1002/path.4205

[57] Li Q , Engebrecht J. BRCA1 and BRCA2 tumor suppressor function in meiosis. Front Cell Dev Biol 2021;9:668309. 33996823 10.3389/fcell.2021.668309 PMC8121103

[58] Kojic M , Kostrub CF, Buchman AR et al BRCA2 homolog required for proficiency in DNA repair, recombination, and genome stability in Ustilago maydis. Mol Cell 2002;10:683–91. 12408834 10.1016/s1097-2765(02)00632-9

[59] Siaud N , Dray E, Gy I et al Brca2 is involved in meiosis in Arabidopsis thaliana as suggested by its interaction with Dmc1. EMBO J 2004;23:1392–401. 15014444 10.1038/sj.emboj.7600146 PMC381417

[60] Wang S , Durrant WE, Song J et al Arabidopsis BRCA2 and RAD51 proteins are specifically involved in defense gene transcription during plant immune responses. Proc Natl Acad Sci U S A 2010;107:22716–21. 21149701 10.1073/pnas.1005978107 PMC3012525

[61] Hopkins BD , Hodakoski C, Barrows D et al PTEN function: the long and the short of it. Trends Biochem Sci 2014;39:183–90. 24656806 10.1016/j.tibs.2014.02.006 PMC4043120

[62] Chen CY , Chen J, He L et al PTEN: tumor suppressor and metabolic regulator. Front Endocrinol 2018;9:338. 10.3389/fendo.2018.00338 PMC6046409 30038596

[63] Pribat A , Sormani R, Rousseau-Gueutin M, Julkowska MM, Testerink C, Joubès J et al A novel class of PTEN protein in Arabidopsis displays unusual phosphoinositide phosphatase activity and efficiently binds phosphatidic acid. Biochem J 2012;441:161–71. 21864294 10.1042/BJ20110776

[64] Vijayakrishnapillai LMK , Desmarais JS, Groeschen MN et al Deletion of ptn1, a PTEN/TEP1 Orthologue, in Ustilago maydis reduces pathogenicity and teliospore development. J Fungi (Basel) [Internet] 2018;5:1. DOI: https://doi.org/10.3390/jof5010001 30577430 PMC6462984

[65] Harbour JW , Dean DC. Rb function in cell-cycle regulation and apoptosis. Nat Cell Biol 2000;2:E65–7. 10783254 10.1038/35008695

[66] Desvoyes B , Gutierrez C. Roles of plant retinoblastoma protein: cell cycle and beyond. EMBO J 2020;39:e105802. 32865261 10.15252/embj.2020105802 PMC7527812

[67] Kojic M , Yang H, Kostrub CF et al The BRCA2-interacting protein DSS1 is vital for DNA repair, recombination, and genome stability in Ustilago maydis. Mol Cell 2003;12:1043–9. 14580353 10.1016/s1097-2765(03)00367-8

[68] Nedelcu AM. The evolution of multicellularity and cancer: views and paradigms. Biochem Soc Trans 2020;48:1505–18. 32677677 10.1042/BST20190992

[69] Caulin AF , Maley CC. Peto’s Paradox: evolution’s prescription for cancer prevention. Trends Ecol Evol 2011;26:175–82. 21296451 10.1016/j.tree.2011.01.002 PMC3060950

[70] Callier V. Solving Peto’s Paradox to better understand cancer. Proc Natl Acad Sci USA 2019;116:1825–8. 30723181 10.1073/pnas.1821517116 PMC6369797

[71] Nedelcu AM. Evo-devo perspectives on cancer. Essays Biochem 2022;66:797–815. 36250956 10.1042/EBC20220041

[72] Hall BK. Evolutionary Developmental Biology (Evo-Devo): past, present, and future. Evol: Educ Outreach 2012;5:184–93.

[73] Bellacosa A. Developmental disease and cancer: biological and clinical overlaps. Am J Med Genet A 2013;161A:2788–96. 24123833 10.1002/ajmg.a.36267 PMC4251736

[74] Laconi E , Marongiu F, DeGregori J. Cancer as a disease of old age: changing mutational and microenvironmental landscapes. Br J Cancer 2020;122:943–52. 32042067 10.1038/s41416-019-0721-1 PMC7109142

[75] López-Otín C , Blasco MA, Partridge L et al The hallmarks of aging. Cell 2013;153:1194–217. 23746838 10.1016/j.cell.2013.05.039 PMC3836174

[76] Aktipis A. Principles of cooperation across systems: from human sharing to multicellularity and cancer. Evol Appl 2016;9:17–36. 27087837 10.1111/eva.12303 PMC4780378

[77] Michod RE , Roze D. Cooperation and conflict in the evolution of multicellularity. Heredity 2001;86:1–7. 11298810 10.1046/j.1365-2540.2001.00808.x

[78] Jackson MDB , Duran-Nebreda S, Bassel GW. Network-based approaches to quantify multicellular development. J R Soc Interface [Internet] 2017;14:20170484. DOI: https://doi.org/10.1098/rsif.2017.0484 29021161 PMC5665831

[79] Celiker H , Gore J. Cellular cooperation: insights from microbes. Trends Cell Biol 2013;23:9–15. 22999189 10.1016/j.tcb.2012.08.010

[80] Niklas KJ , Newman SA. The origins of multicellular organisms. Evol Dev 2013;15:41–52. 23331916 10.1111/ede.12013

[81] Herron MD , Rashidi A, Shelton DE et al Cellular differentiation and individuality in the “minor” multicellular taxa. Biol Rev Camb Philos Soc 2013;88:844–61. 23448295 10.1111/brv.12031 PMC4103886

[82] Rokas A. The origins of multicellularity and the early history of the genetic toolkit for animal development. Annu Rev Genet 2008;42:235–51. 18983257 10.1146/annurev.genet.42.110807.091513

[83] Ispolatov I , Ackermann M, Doebeli M. Division of labour and the evolution of multicellularity. Proc Biol Sci 2012;279:1768–76. 22158952 10.1098/rspb.2011.1999 PMC3297448

[84] Erten EY , Kokko H. From zygote to a multicellular soma: body size affects optimal growth strategies under cancer risk. Evol Appl [Internet] 2020;13:1593. https://onlinelibrary.wiley.com/doi/abs/10.1111/eva.12969.

[85] Boddy AM , Kokko H, Breden F et al Cancer susceptibility and reproductive trade-offs: a model of the evolution of cancer defences. Philos Trans R Soc Lond B Biol Sci [Internet] 2015;370:20140220. DOI: https://doi.org/10.1098/rstb.2014.0220 26056364 PMC4581025

[86] Seluanov A , Gladyshev VN, Vijg J et al Mechanisms of cancer resistance in long-lived mammals. Nat Rev Cancer 2018;18:433–41. 29622806 10.1038/s41568-018-0004-9 PMC6015544

[87] Tollis M , Ferris E, Campbell MS et al Elephant genomes reveal accelerated evolution in mechanisms underlying disease defenses. Mol Biol Evol [Internet] 2021;38:3606–20. DOI: https://doi.org/10.1093/molbev/msab127 33944920 PMC8383897

[88] Fisher GJ. Cancer resistance, high molecular weight hyaluronic acid, and longevity. J Cell Commun Signal 2015;9:91–2. 25740467 10.1007/s12079-015-0278-6 PMC4414831

[89] Tinbergen N. On aims and methods of Ethology. Z Tierpsychol 2010;20:410–33.

[90] Nesse RM. Tinbergen’s four questions, organized: a response to Bateson and Laland. Trends Ecol Evol 2013;28:681–2. 24216179 10.1016/j.tree.2013.10.008

[91] de Magalhães JP. Every gene can (and possibly will) be associated with cancer. Trends Genet 2022;38:216–7. 34756472 10.1016/j.tig.2021.09.005

[92] Kowalczyk A , Partha R, Clark N et al *Cancer Control Is a Key Functionality Underlying Evolution of Extended Lifespan in Mammals [Internet]*. bioRxiv. 2019. p. 615914. https://www.biorxiv.org/content/10.1101/615914v1.abstract (29 June 2023, date last accessed).

[93] Gorbunova V , Seluanov A, Zhang Z et al Comparative genetics of longevity and cancer: insights from long-lived rodents. Nat Rev Genet 2014;15:531–40. 24981598 10.1038/nrg3728 PMC4353926

[94] Williams GC. A defense of reductionism in evolutionary biology. Oxf Surv Evol Biol 1985;2:1–97.

